# Microfluidic Production of Dexamethasone-Loaded PLGA Microparticles: Dynamic Solvent Extraction Improves Process Robustness During the Droplet-to-Particle Transition

**DOI:** 10.3390/pharmaceutics18091075

**Published:** 2026-08-27

**Authors:** Nader Amanatchi, Ilyesse Bihi, Matthieu Briet, Wim De Malsche, Karine H. Hellemans

**Affiliations:** 1 Unit Diabetes Pathology and Therapy, Faculty of Medicine and Pharmacy, Vrije Universiteit Brussel, Laarbeeklaan 103, 1090 Brussels, Belgium; 2 µFlow Group, Department of Chemical Engineering, Vrije Universiteit Brussel, Pleinlaan 2, 1050 Brussels, Belgium

**Keywords:** PLGA, microfluidics, process reproducibility, inter-batch variability, particle morphology, solvent extraction, microparticle fabrication, sustained drug release, release kinetics, dexamethasone

## Abstract

**Background/Objectives:** Poly(lactic-co-glycolic acid) (PLGA) microparticles are used for sustained drug delivery, yet their final quality depends on both initial droplet formation and the subsequent solvent-extraction-driven droplet-to-particle transition. Microfluidics provides excellent control over precursor droplets, but this control may be partially lost during particle formation. We previously developed dynamic solvent extraction (DSE), in which the droplet-to-particle transition occurs progressively during continuous transport through an extended microfluidic channel. Here, we investigated whether this control improves quality attributes and release of drug-loaded PLGA microparticles. **Methods:** Dexamethasone (DEX)-loaded PLGA microparticles were produced using DSE or static solvent extraction (SSE), in which microfluidic droplets were transferred to an external aqueous medium for particle formation. Three PLGA concentrations (2.5, 5, and 10% (*w*/*v*)) were investigated, with processing conditions selected to obtain comparable final particle sizes and reduce size as a confounding variable. **Results:** DSE significantly reduced particle-size coefficient of variation compared with SSE, indicating better preservation of size uniformity. Drug loading and encapsulation efficiency were governed mainly by PLGA concentration, with no significant effect of extraction strategy. Morphological effects were formulation-dependent, with the most pronounced defects in 5% (*w*/*v*) SSE particles. All formulations provided sustained DEX release over 70 days. Although the overall effect of extraction strategy on 24 h burst release was not statistically significant, DSE consistently produced numerically lower burst release, with the largest and most variable burst observed for the morphologically heterogeneous 5% (*w*/*v*) SSE formulation. **Conclusions:** These findings extend DSE to drug-loaded sustained-release microparticles and identify the solvent-extraction environment as an important determinant of final particle quality.

## 1. Introduction

Many therapeutic agents require sustained exposure at the target site to achieve optimal efficacy. Poly(lactic-co-glycolic acid) (PLGA) is one of the most widely used biodegradable polymers for sustained drug delivery because of its excellent safety profile, biocompatibility, and tunable degradation kinetics, enabling drug release over periods ranging from hours to several months [1,2,3]. Although several long-acting PLGA-based formulations have been approved by the U.S. Food and Drug Administration (FDA) and the European Medicines Agency (EMA), relatively few products have successfully reached the market [4,5,6]. One of the major challenges remains the reproducible manufacture of microparticles with predictable release characteristics. In particular, the initial burst release and subsequent lag phase frequently observed in PLGA microspheres remain difficult to control [7,8,9,10]. These release characteristics are strongly influenced by critical quality attributes (CQAs) of the microparticles, including particle size, morphology, drug loading (DL), and polymer distribution [11,12,13,14]. Consequently, reproducible control over microparticle formation is essential to achieve consistent therapeutic performance. However, conventional emulsification techniques provide limited control over both droplet formation and the subsequent solvent-extraction-driven particle formation process, which can contribute to substantial batch-to-batch variability in these CQAs [15,16,17,18,19].

Microfluidic droplet generation offers a highly controlled alternative to conventional emulsification by producing individual droplets under precisely defined flow conditions [20]. Among the available droplet-generation geometries, co-flow systems are particularly suitable for oil-in-water emulsions containing viscous polymer solutions. In this configuration, the dispersed phase is confined within the center of the continuous phase, minimizing contact with the channel walls and thereby reducing polymer fouling while enabling highly reproducible droplet formation [21,22]. However, improved control of droplet generation addresses only the initial stage of microparticle manufacture. Formation of monodisperse precursor droplets does not necessarily ensure equivalently monodisperse final microparticles because substantial physicochemical transformation continues during solvent extraction. Solvent exchange induces polymer phase separation and precipitation, particle shrinkage, surface-layer formation, and progressive transformation of the liquid precursor droplet into a formed microparticle. These processes depend strongly on local mass-transfer conditions, and variations in solvent exchange can alter polymer precipitation and particle structure, ultimately affecting microparticle morphology and performance [23,24,25]. The solvent-extraction-driven droplet-to-particle transition therefore represents a second critical stage at which the control achieved during microfluidic droplet generation may either be preserved or lost.

Recent developments in microfluidics have extended process control beyond droplet generation by enabling solvent extraction to proceed during continuous transport through structured microchannels [26,27,28]. In our previous work, we developed a dynamic solvent extraction (DSE) platform in which freshly generated PLGA droplets undergo progressive solvent extraction and shrinkage during transport through an extended serpentine microchannel [26]. This study established the physicochemical basis of DSE and demonstrated controlled droplet-to-particle transformation for blank PLGA microparticles. The pharmaceutical relevance of controlling this process step, however, remains to be established. In particular, it is unknown whether the process control provided by DSE is retained upon drug incorporation and translates into improved critical quality attributes and more reproducible release behavior. Direct comparisons with static solvent extraction (SSE), in which microfluidically generated droplets undergo the major part of their subsequent transformation after transfer to a bulk aqueous phase, are also scarce. Consequently, it remains unclear whether precise control of precursor droplet formation alone is sufficient to ensure reproducible drug-loaded microparticles, or whether control of the subsequent solvent-extraction environment provides an additional determinant of final product quality.

To address this question, the present study directly compared DSE and SSE for the production of dexamethasone (DEX)-loaded PLGA microparticles across three polymer concentrations (2.5, 5, and 10% (*w*/*v*)). DEX was selected as a pharmaceutically relevant model compound for sustained PLGA delivery. DEX is a potent anti-inflammatory glucocorticoid that has been widely investigated for sustained delivery from PLGA-based systems, providing a pharmaceutically relevant model for evaluating drug incorporation and release [29,30]. For each PLGA concentration, both extraction strategies employed the same co-flow droplet-generation principle and identical formulation composition, while formulation-specific processing conditions were empirically selected to obtain microparticles within a comparable final size range and thereby minimize particle size as a confounding variable. Particle-size distribution and batch-to-batch reproducibility, drug loading, encapsulation efficiency, morphology, and 70-day in vitro DEX release were subsequently evaluated. By separating control of precursor droplet generation from control of the subsequent solvent-extraction environment, this study examines whether DSE can preserve microfluidic process control through the droplet-to-particle transition and thereby improve the reproducible manufacture of drug-loaded sustained-release PLGA microparticles.

## 2. Materials and Methods

### 2.1. Chemicals

Poly(D,L-lactide-co-glycolide) (PLGA; acid-terminated, 75:25, Mw 4–15 kDa; RESOMER^®^ RG 752H), ethyl acetate (EA; anhydrous, 99.8%), dexamethasone (DEX; BioReagent, suitable for cell culture, ≥97%), polyvinyl alcohol (PVA; 80% hydrolyzed), cyclohexane (anhydrous, ≥99%), methanol (MeOH; 99.9%, HPLC grade), and dimethyl sulfoxide (DMSO; 99.9%, HPLC grade) were purchased from Sigma-Aldrich/Merck (Darmstadt, Germany). Acrylamide/bis-acrylamide solution (40%, 29:1), polyacrylamide, and Pluronic^®^ F-68 were obtained from Sigma-Aldrich/Merck (Darmstadt, Germany). Ethanol (≥99.5%) and 2-propanol were purchased from VWR International (Leuven, Belgium). Phosphate-buffered saline (PBS; TheraPEAK^®^ PBS, without calcium or magnesium, GMP grade) was obtained from Lonza (Walkersville, MD, USA).

### 2.2. Microfluidic Chip Design

**Figure 1:**
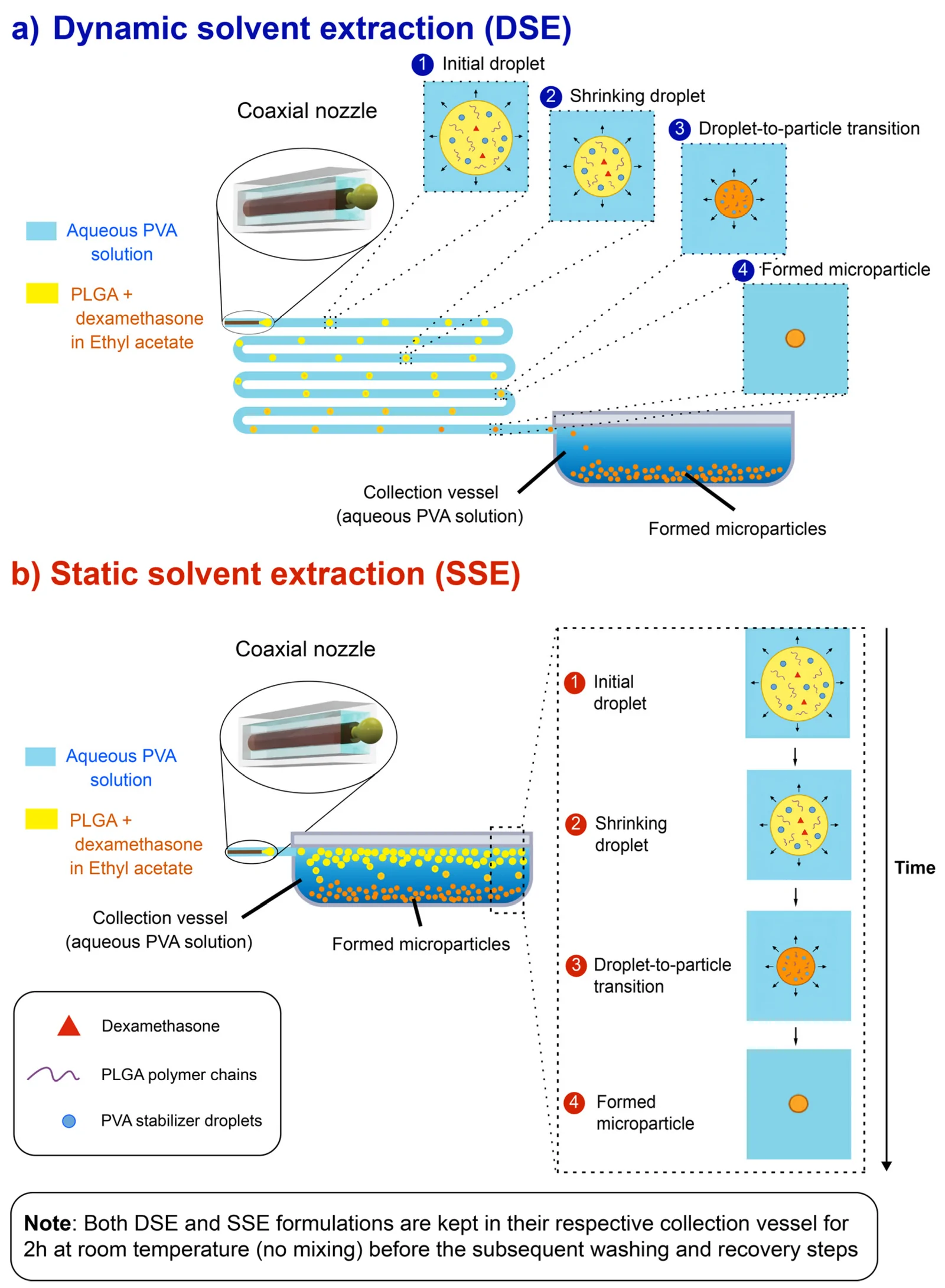
Schematic comparison of dynamic solvent extraction (DSE) and static solvent extraction (SSE) during PLGA microparticle fabrication. In both approaches, droplets containing PLGA and DEX dissolved in ethyl acetate are generated using a co-flow microfluidic nozzle within an aqueous PVA continuous phase. (**a**) In DSE, droplets flow through an extended microfluidic channel that enables progressive solvent extraction, droplet shrinkage, and the droplet-to-particle transition prior to collection, resulting in formed microparticles. (**b**) In SSE, droplets are transferred directly into an aqueous PVA collection vessel, where solvent extraction, droplet shrinkage, and the droplet-to-particle transition occur predominantly under static off-chip conditions. The numbered illustrations (1–4) schematically represent the progression from the initial droplet through droplet shrinkage and the droplet-to-particle transition to the formed microparticle. In both DSE and SSE, the samples were kept in aqueous PVA collection vessels for 2 h at room temperature without stirring and subsequently washed and recovered. For DSE, solvent extraction occurs progressively during on-chip transport before collection, whereas for SSE, most of the solvent extraction and droplet-to-particle transition occur within the external collection medium.

The experiments were performed using a cyclic olefin copolymer grade 6013 (COC 6013) microfluidic platform based on the device previously developed by Amanatchi et al. [26]. The COC 6013 plates were obtained from Kunststoff-Zentrum in Leipzig GmbH (Leipzig, Germany). The device had a coaxial droplet-generation region followed by a downstream serpentine channel used for solvent extraction and the subsequent droplet-to-particle transition. The dispersed and continuous phases were introduced separately through two inlet channels. Each channel was 500 µm wide and 500 µm deep. These channels merged into a larger square channel cross-section of 800 × 800 µm, which remained uniform throughout the entire downstream serpentine section (Figure 1).

Droplet generation was achieved using a coaxial capillary configuration. A fused-silica capillary (200 µm ID, 360 µm OD; Polymicro Technologies, Phoenix, AZ, USA) carrying the dispersed phase, consisting of PLGA and DEX in ethyl acetate, was inserted through one inlet and positioned concentrically within the 800 × 800 µm channel. The aqueous PVA continuous phase was introduced through the second inlet and flowed around the exterior of the capillary. The dispersed and continuous phases were delivered independently using two syringe pumps (SP100IZ, World Precision Instruments, Sarasota, FL, USA), allowing their respective flow rates to be controlled separately. At the capillary outlet, the dispersed phase was surrounded by the flowing continuous phase, resulting in droplet formation. The dispersed-phase (Q_d_) and continuous-phase (Q_c_) flow rates were controlled independently according to the specific operating conditions used for microparticle preparation described in Section 2.5.

For dynamic solvent extraction (DSE), after the droplet-generation region, an 80 cm long serpentine channel in COC 6013 was employed, with a uniform square cross-section 800 × 800 µm for the entire length. The serpentine consisted of straight sections connected by curved U-shaped turns. The extended flow path enabled sufficient residence time for progressive extraction of ethyl acetate from individual droplets into the continuously flowing aqueous PVA phase, thereby enabling progressive droplet shrinkage and the droplet-to-particle transition during downstream transport. Droplet formation and shrinkage were visually monitored at multiple locations along the serpentine channel to observe the transformation of freshly generated droplets into formed microparticles; no pre-designated quantitative observation locations were used. The internal volume of the DSE device was approximately 520 µL.

For static solvent extraction (SSE), a second microfluidic device with the same local coaxial nozzle geometry as in Figure 1a was used to yield comparable droplet-generation conditions (Figure 1b). Here, the downstream channel was shortened to approximately 1 cm and connected to a 2 cm long fused-silica outlet capillary (350 μm ID, 670 μm OD; Polymicro Technologies, Phoenix, AZ, USA), resulting in a reduced internal volume of approximately 6.4 μL of the microfluidic assembly. In this way, the residence time of freshly generated droplets within the device was minimized to limit solvent extraction and droplet shrinkage before their collection. Thereafter, freshly generated droplets were continuously transferred into a bulk aqueous PVA solution where ethyl acetate extraction, progressive droplet shrinkage, and the droplet-to-particle transition occurred predominantly under static conditions.

Both configurations employed the same coaxial droplet-generation principle, but differed in the environment in which solvent extraction and the droplet-to-particle transition predominantly occurred. In DSE, these processes occurred dynamically during continuous downstream transport through the extended serpentine channel of the device. Conversely, in the SSE configuration, freshly generated droplets were rapidly transferred to the bulk collection medium, where most subsequent solvent extraction and droplet-to-particle transition occurred.

### 2.3. Microfluidic Chip Fabrication

The fabrication process involved two steps. First, the channels were micromilled with a DATRON neo CNC milling machine (DATRON AG, Mühltal, Germany) on a COC layer. Subsequently, the micromilled COC layer was bonded to the COC lid plate using the solvent bonding method [31]. Briefly, both COC layers were brushed with soapy water, rinsed with deionized water, degreased with isopropanol, and air-dried before the bonding process. Visual inspection was essential to ensure that there were no residues remaining from the milling process that could potentially block the channels. The lid layer was then exposed to cyclohexane for 4 min at a distance of 1 cm. The chip was then assembled, layers positioned on top of each other, and a thermocompression of 5 kN at 70 °C for 5 min was applied. Finally, capillaries were glued to the chip. To form the coaxial nozzle, the dispersed phase, a circular fused-silica capillary with an inner diameter (ID) of 200 µm and an outer diameter of 360 µm, was inserted through the inlet channel until reaching the serpentine channel.

### 2.4. Chip Coating

The COC channel surface is inherently hydrophobic. To prevent droplets from wetting the surface, we applied a hydrophilic coating on the chip according to the protocol described by [32]. Briefly, a hydrophilic coating solution was prepared by mixing 45 mL of 40 % acrylamide solution, 2 mL of benzophenone/ethanol solution, 2 mL of Pluronic^®^ F-68, and 11 mL of Milli-Q^®^ water to achieve a total volume of 60 mL. The benzophenone was first dissolved in ethanol at a concentration of 0.1 mg/mL. After purging the solution with nitrogen gas for 5 min, the solution was pumped into the serpentine channel through the continuous phase inlet. Subsequently, the reaction was mediated by ultraviolet (UV) light exposure at a wavelength of 254 nm for 30 min. After coating, the channels were flushed with deionized water, then dried with pressurized air.

### 2.5. Preparation of DEX-Loaded PLGA Microparticles

DEX-loaded PLGA microparticles were prepared using a microfluidic droplet-based approach. The dispersed phase consisted of PLGA (2.5, 5, and 10% (*w*/*v*)) dissolved in ethyl acetate. These concentrations represented successive two-fold increases in polymer concentration, providing a systematic four-fold range over which the influence of PLGA concentration was evaluated. Corresponding DEX concentrations of 5, 10, and 20 mg/mL were used, respectively, thereby maintaining a constant DEX:PLGA mass ratio of 1:5 across all formulations. The continuous phase consisted of a 5% (*w*/*v*) PVA aqueous solution. For all formulations, the dispersed-phase flow rate was fixed at Q_d_ = 0.12 mL/h, while the continuous-phase flow rate was set to Q_c_ = 5, 10, and 20 mL/h for the 2.5, 5, and 10% (*w*/*v*) PLGA formulations, respectively. The chosen formulation-specific flow rates of the continuous phase were selected to yield a comparable final particle diameter across the different PLGA concentrations by counterbalancing the differences in precursor droplet size and subsequent shrinkage during solvent extraction. The lower PLGA concentrations required a larger precursor droplet because of the greater extent of shrinkage, whereas the higher PLGA concentrations required a smaller precursor droplet to obtain comparable final particle diameters.

After collection, DSE and SSE microparticles were maintained in their aqueous PVA collection media for 2 h at room temperature under static conditions (no stirring or other agitation). The microparticles were collected with a glass Pasteur pipette and transferred to 4 mL tubes. Particles were washed three times with Milli-Q water by centrifugation to remove residual PVA and solvent. The final pellet was air-dried for at least 8 h before storage at −20 °C until further characterization.

### 2.6. Microparticle Size and Morphology Analysis

Particle size was determined by light microscopy using a Nikon Eclipse Ti microscope (Nikon, Tokyo, Japan). The particle diameters were determined from microscopy images obtained using NIS-Elements software (version 4.50; Nikon, Tokyo, Japan). A minimum of 50 individual microparticles was measured per independent production batch. The mean particle diameter and standard deviation (SD) were calculated per batch. Particle-size variability was expressed as the coefficient of variation (CV), calculated according to Equation (1):

(1) $$CV\;\left(\%\right)=(\frac{SD}{mean\;particle\;diameter}\;)\times 100.$$

The microparticle morphology was analyzed by scanning electron microscopy (SEM) using a JEOL JSM-IT300 (JEOL Ltd., Tokyo, Japan). Dried microparticles were mounted onto SEM specimen stubs using double-sided adhesive tape (Science Services GmbH, Munich, Germany) and sputter-coated with a 3 nm gold layer prior to imaging. Imaging was performed under high-vacuum conditions using a secondary electron detector (SED). Depending on the magnification and imaging purpose, accelerating voltages (5–20 kV) and working distances of approximately 10.1–10.4 mm were used. Images were acquired at magnifications ranging from 150× to 2000×. For each PLGA concentration and solvent-extraction strategy, microparticles from three independent production batches were examined by SEM. For each batch, multiple fields were examined at low magnification to assess overall particle morphology and the presence of morphological heterogeneity or structural defects. Higher-magnification imaging was subsequently performed to characterize representative surface features and, where present, localized structural defects.

### 2.7. Drug Loading and Encapsulation Efficiency

Drug loading (DL) was evaluated by ultraviolet–visible (UV–Vis) spectrophotometry following dissolution of DEX-loaded microparticles. Approximately 3 mg of dried microparticles from each independent production batch were accurately weighed and dissolved in 300 µL DMSO to ensure complete dissolution of the polymer matrix and release of encapsulated DEX. No further dilution or sample processing was performed. Absorbance was measured at 252 nm using a NanoDrop ND-1000 UV–Vis spectrophotometer (Thermo Fisher Scientific, Wilmington, DE, USA), with DMSO used as the blank. DEX concentration was determined from a calibration curve prepared in DMSO over 10–4000 µg/mL (25–10,000 µM), which showed a linear response over this range (y = 0.0241x + 0.3762; R^2^ = 0.9968). The experimentally determined DEX concentration and dissolution volume were used to calculate the mass of DEX in the analyzed microparticle sample. DL was calculated according to Equation (2), consistent with previously reported approaches for DEX-loaded PLGA microparticles [33]:

(2) $$DL\;\left(\%\right)=(\frac{m_{DEX,particles}}{m_{particles}})\times 100.$$

For calculation of encapsulation efficiency (EE), the initial drug loading (IDL) was determined separately for each production batch from the actual masses of DEX and PLGA used during preparation, according to Equation (3):

(3) $$IDL\;\left(\%\right)=(\frac{m_{DEX,initial}}{m_{DEX,initial}+m_{PLGA,initial}})\times 100.$$

EE was calculated as the experimentally determined DL relative to the corresponding batch-specific IDL, according to Equation (4):

(4) $$EE\;\left(\%\right)=(\frac{DL\;(\%)}{IDL\;(\%)})\times 100.$$

This approach is consistent with previously reported calculations of encapsulation efficiency for DEX-loaded PLGA microparticles [34].

### 2.8. In Vitro Release Kinetics

In vitro DEX release was evaluated under static conditions in PBS (pH 7.4) at 37 °C. PBS at pH 7.4 was selected to provide physiologically relevant aqueous conditions and has previously been used as a release medium for DEX-loaded PLGA microparticles [29,35]. To ensure that all formulations exhibited comparable initial amounts of DEX, the mass of microparticles used in release experiments was adjusted based on the experimentally determined DL, resulting in microparticle masses of approximately 3–5 mg. The PBS volume was then individually adjusted between 3 and 15 mL such that the theoretical DEX concentration measured at each time point would be within the linear range of the analytical calibration curve (2.5–120 µM).

Microparticles were suspended in PBS and incubated at 37 °C without agitation to minimize mechanical disruption of the particles during long-term release. Samples were collected after 1, 2, 4, 6, 8, 10, 12, 14, 21, 35, 57 and 70 days. At each sampling point, 100 µL of release medium was collected and replaced by an equal volume of fresh PBS to maintain a constant release volume throughout the experiment.

The concentration of DEX was quantified using the NanoDrop ND-1000 at 242 nm using calibration standards prepared in PBS with 0.2% (*v*/*v*) DMSO over a concentration range of 2.5–120 µM. Prior to analysis, DMSO was added to the sampled release medium to obtain a final DMSO concentration of 0.2% (*v*/*v*), corresponding to the matrix used for calibration.

Cumulative DEX release was determined as a percentage of the total encapsulated DEX. It was corrected for the effects of serial sampling and medium replacement. Release studies were carried out using three independent production batches for each formulation, except for the 10% (*w*/*v*) SSE formulation, for which four independent production batches were analyzed.

### 2.9. Statistical Analysis

Data are presented as mean ± standard deviation (SD) unless otherwise stated. Independent production batches were considered experimental replicates. The effects of polymer concentration and solvent-extraction strategy on particle-size variability, drug loading, encapsulation efficiency and burst release were evaluated using two-way analysis of variance (ANOVA). Where multiple comparisons were required, Šídák’s post hoc test was applied to account for multiple testing. Statistical significance was defined as *p* < 0.05.

Boxplots are presented as the median and interquartile range (Q1–Q3), with whiskers representing the full data range and individual points corresponding to independent production batches. Release-model fitting was performed separately for each independent batch, and the resulting model parameters and goodness-of-fit values were subsequently summarized as mean ± SD. Statistical analyses were performed using GraphPad Prism version 10.2.2 (GraphPad Software, Boston, MA, USA).

Cumulative DEX release profiles were analyzed using DDSolver version 1.0, an add-in for Microsoft Excel for Microsoft 365 (version 2608; Microsoft Corporation, Redmond, WA, USA), which was used for dissolution and drug release data analysis [36]. The release profiles from each batch were fitted by nonlinear regression to zero-order, first-order, Higuchi, Hixson–Crowell, Korsmeyer–Peppas, and Weibull models. The performance of the model was evaluated using an adjusted coefficient of determination (R^2^adj). The release exponent (n) was additionally determined for the Korsmeyer–Peppas model.

## 3. Results and Discussion

### 3.1. Experimental Design Isolates the Effect of the Solvent-Extraction Environment

**Figure 2:**
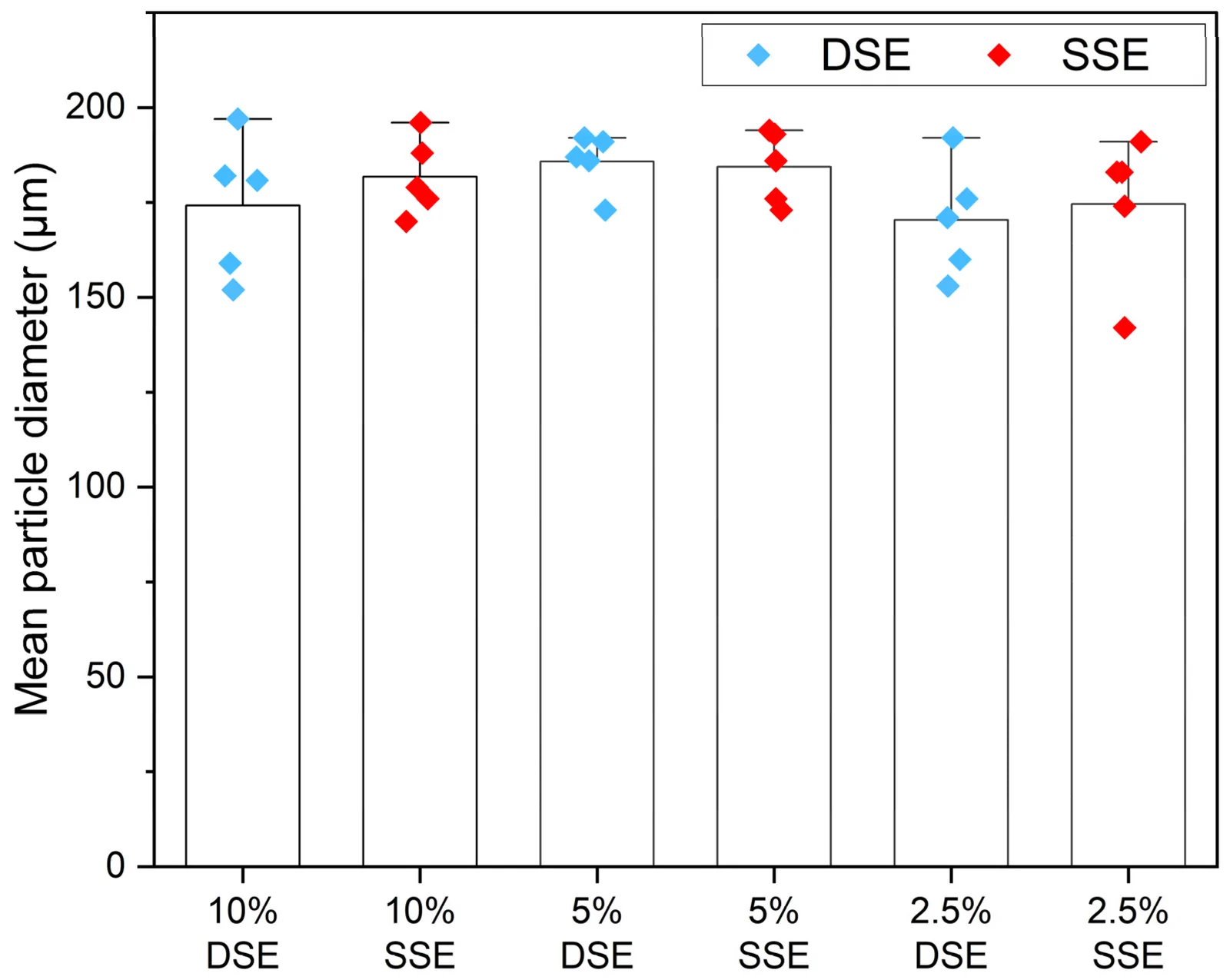
Final particle size of DEX-loaded PLGA microparticles produced by dynamic solvent extraction (DSE) and static solvent extraction (SSE). Mean particle diameters were determined for microparticles prepared at 10, 5, and 2.5% (*w*/*v*) PLGA. Process parameters were adjusted for each PLGA concentration to compensate for concentration-dependent droplet shrinkage and obtain comparable final particle sizes across formulation and solvent-extraction conditions. Bars represent the mean ± standard deviation (SD), and individual symbols represent independent production batches (n = 5). Statistical analysis was performed using two-way ANOVA with PLGA concentration and solvent-extraction strategy as factors. No significant effects of PLGA concentration (*p* = 0.1551), solvent-extraction strategy (*p* = 0.5048), or their interaction (*p* = 0.7712) on mean particle diameter were detected.

DEX-loaded PLGA microparticles were produced at three PLGA concentrations (2.5, 5, and 10% (*w*/*v*)) using dynamic solvent extraction (DSE) or static solvent extraction (SSE). Formulation-specific continuous-phase flow rates were selected to obtain comparable final particle sizes across PLGA concentrations and thereby minimize particle size as a confounding variable when evaluating the effects of solvent-extraction strategy. This was particularly important because particle size can independently influence release behavior through differences in surface-area-to-volume ratio and diffusion path length, and comparison of formulations at matched particle size is therefore recommended when release characteristics are evaluated [37]. As shown in Figure 2, the formulation-specific size-matching conditions resulted in comparable final particle diameters across the investigated conditions. Two-way ANOVA showed no significant effect of PLGA concentration (*p* = 0.1551) or solvent-extraction strategy (*p* = 0.5048) on mean particle diameter, and no significant interaction between these factors (*p* = 0.7712).

The different PLGA concentrations and associated size-matching flow conditions nevertheless resulted in different timescales for the droplet-to-particle transition during DSE. As demonstrated previously for this microfluidic platform, increasing the PLGA concentration reduces the overall extent of droplet shrinkage required to reach the final particle diameter, while the shrinkage rate was largely independent of PLGA concentration when evaluated under identical flow conditions [26]. This concentration-dependent difference in shrinkage is consistent with previous reports showing that lower polymer concentrations permit greater droplet shrinkage during solvent removal, particularly in ethyl-acetate-based systems [37]. Consequently, higher-PLGA droplets reached their final dimensions earlier because a smaller overall reduction in diameter was required. In the present study, this difference was further accentuated by the formulation-specific continuous-phase flow rates used for size matching, with higher continuous-phase flow rates applied at increasing PLGA concentrations. Consequently, the 10% (*w*/*v*) PLGA formulation reached a stable particle diameter over the shortest residence time, followed by the 5% and 2.5% (*w*/*v*) formulations. Overall, dimensional stabilization during DSE occurred within several tens of seconds to approximately 2 min, depending on the formulation and associated flow conditions.

In contrast, during SSE, the freshly generated droplets were promptly transferred into the external collection phase, where solvent extraction, droplet shrinkage, and droplet-to-particle transition took place mostly in the bulk and over a much longer timescale. By keeping the final particle size approximately the same for all formulations, and maintaining a constant DEX-to-PLGA ratio across all formulations, differences arising from final particle size and relative initial drug content were minimized. Thus, the experimental design permitted the effect of the solvent-extraction environment on the properties of the resulting microparticles to be assessed with reduced confounding from these variables.

### 3.2. Dynamic Solvent Extraction Preserves Particle-Size Uniformity Across Production Batches

**Figure 3:**
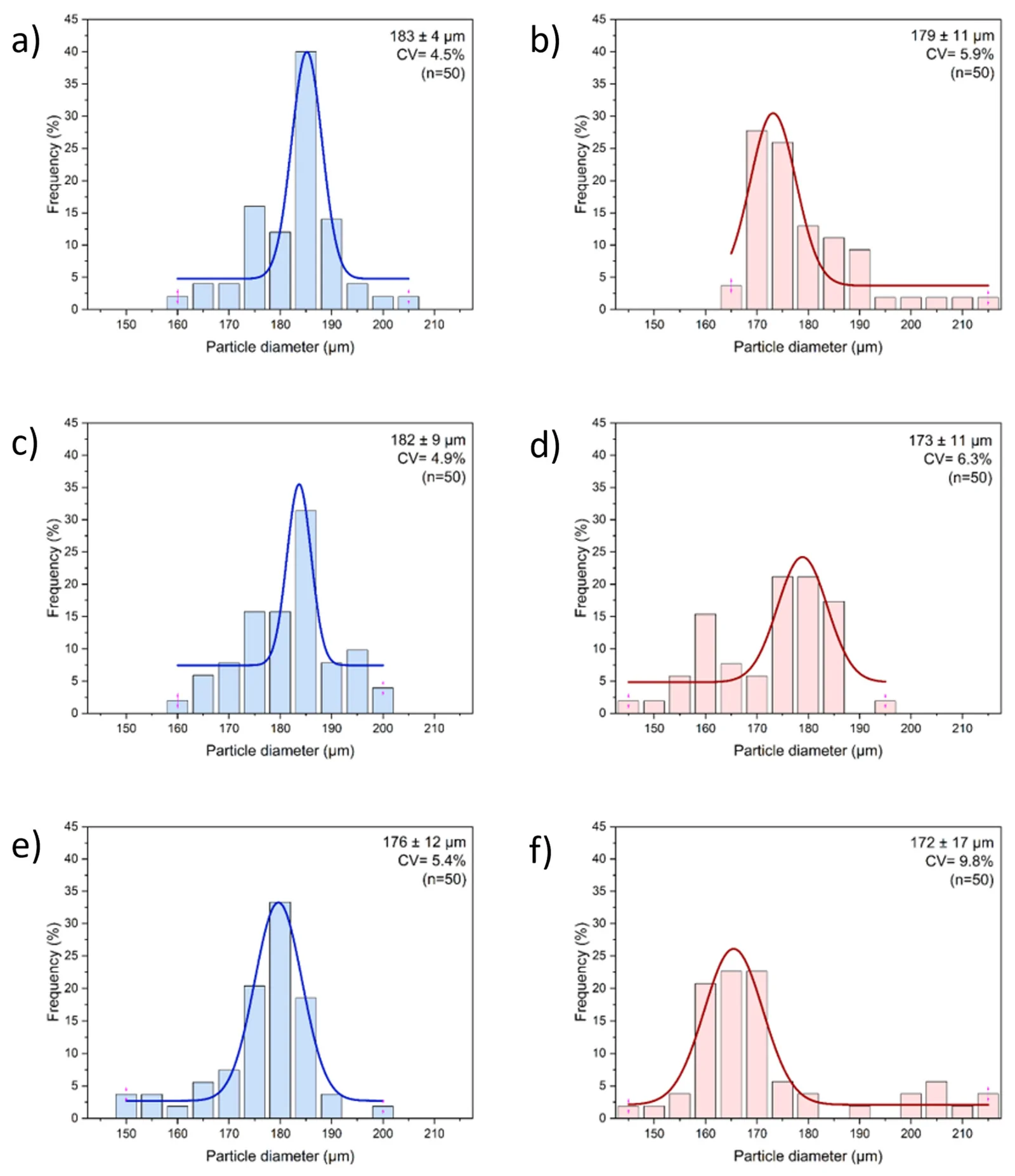
Representative particle size distributions of DEX-loaded PLGA microparticles produced by dynamic solvent extraction (DSE) and static solvent extraction (SSE) at three PLGA concentrations. Histograms show the within-batch frequency distribution of final particle diameters for representative production batches, with the superimposed curves indicating the fitted distributions. Panels correspond to 10% (*w*/*v*) PLGA DSE (**a**), 10% (*w*/*v*) PLGA SSE (**b**), 5% (*w*/*v*) PLGA DSE (**c**), 5% (*w*/*v*) PLGA SSE (**d**), 2.5% (*w*/*v*) PLGA DSE (**e**), and 2.5% (*w*/*v*) PLGA SSE (**f**). Mean particle diameter ± standard deviation (SD), coefficient of variation (CV), and the number of particles analyzed (n) are indicated within each panel.

Having established comparable final particle sizes across formulations and solvent-extraction strategies, we next examined whether particle-size uniformity differed between DSE and SSE. Representative size distribution histograms (Figure 3) illustrate how DSE produces a narrower particle size distribution than SSE for all PLGA concentrations, while still exhibiting comparable mean particle diameter. This difference was most pronounced for the 2.5% (*w*/*v*) formulation, where SSE generated the broadest distribution. These representative data suggest that DSE better preserves the size uniformity established during droplet generation throughout the subsequent droplet-to-particle transition.

**Figure 4:**
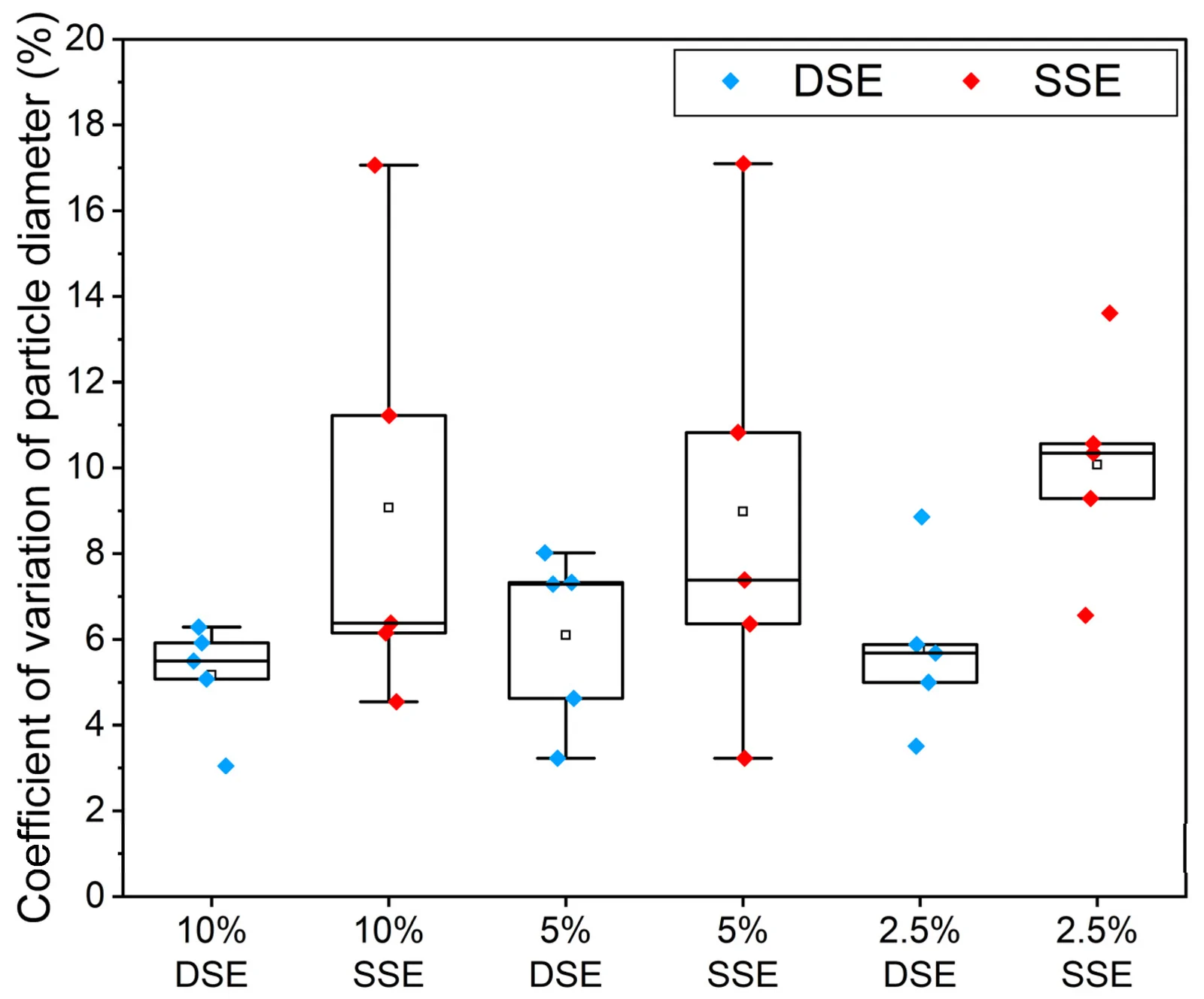
Effect of solvent-extraction strategy and polymer concentration on the particle-size distribution. The coefficient of variation (CV) of particle diameter was determined for microparticles prepared by DSE and SSE at polymer concentrations of 10, 5, and 2.5% (*w*/*v*). Each symbol represents an independent production batch (n = 5). Boxes indicate the interquartile range, the central line represents the median, and whiskers show the full data range. Lower CV values indicate a narrower particle size distribution and improved particle monodispersity. The distribution of individual batches provides insight into the reproducibility and robustness of particle production under each processing condition.

To examine whether this observation was consistent across independent production batches, the coefficient of variation (CV) was analyzed for all batches (Figure 4). DSE presented lower CV values than SSE across all three PLGA concentrations. Two-way ANOVA showed a significant effect of solvent-extraction strategy on particle size CV (*p* = 0.0069), whereas polymer concentration had no significant effect (*p* = 0.870), and no interaction between the two factors was detected (*p* = 0.894). These results indicate that particle-size uniformity within the investigated formulation range was significantly associated with solvent-extraction strategy, whereas no significant effect of polymer concentration was detected. The distribution of batch-level CV values further differed between formulations: SSE showed pronounced batch-to-batch variation at 10% and 5% (*w*/*v*) PLGA, whereas the 2.5% (*w*/*v*) SSE formulation exhibited consistently elevated CV values with a comparatively narrow interquartile range (Figure 4, Table 1).

The findings demonstrate that the monodispersity established during microfluidic droplet generation is not necessarily preserved during the subsequent droplet-to-particle transition. Kong et al. showed similar results in which initially monodisperse PLGA emulsion droplets can give rise to much broader final particle-size distributions when particle formation subsequently occurs under less controlled extraction conditions [25]. On the other hand, microfluidic solvent-extraction approaches have been reported to produce highly monodisperse PLGA particles when solvent removal occurs under well-defined conditions [28]. In our previous work using the present microfluidic platform, DSE resulted in reproducible droplet-shrinkage trajectories and more monodisperse final PLGA microparticles compared with SSE [26]. The present findings extend these observations to DEX-loaded microparticles and show that the solvent-extraction strategy significantly affects preservation of particle-size uniformity across independent production batches. Together, these observations are consistent with the idea that confining the droplet-to-particle transition within a well-defined flow path enhances control over solvent extraction and shrinkage, and consequently better preserves size uniformity established during droplet generation.

Beyond the overall effect on particle-size CV, the distribution of individual batches revealed distinct patterns of process variability between formulations (Figure 4; Table 1). The 10% and 5% (*w*/*v*) SSE formulations showed broad distributions of batch-level CVs, including individual batches with markedly elevated values, indicating greater batch-to-batch variation in preservation of particle-size uniformity. In contrast, the 2.5% (*w*/*v*) SSE formulation showed a consistently elevated median CV relative to DSE (10.45% versus 5.68%) but a comparatively narrow interquartile range. Thus, for the 2.5% formulation, the broader within-batch particle-size distribution was reproducibly observed across independent production batches rather than being driven by isolated poorly performing batches. This is consistent with the broader representative 2.5% (*w*/*v*) SSE distribution shown in Figure 3f.

### 3.3. Drug Loading and Encapsulation Efficiency Are Primarily Governed by PLGA Concentration Rather than Solvent-Extraction Strategy

**Figure 5:**
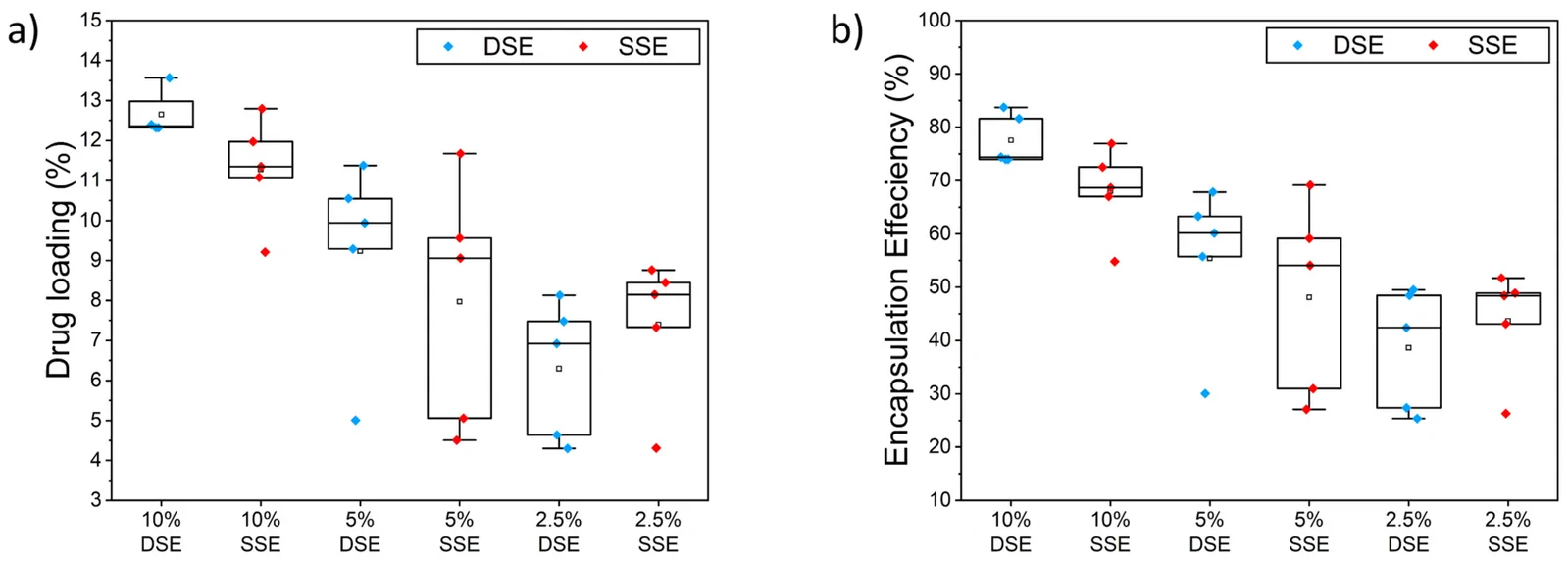
Effect of solvent-extraction strategy and PLGA concentration on the performance of DEX-loaded microparticles produced by dynamic solvent extraction (DSE) and static solvent extraction (SSE). (**a**) Drug loading and (**b**) encapsulation efficiency were determined for particles prepared by DSE and SSE at polymer concentrations of 10, 5, and 2.5% (*w*/*v*). Each symbol represents an independent batch (n = 5). Boxes indicate the interquartile range, the central line represents the median, and whiskers show the full data range. Statistical analysis was performed using two-way ANOVA with PLGA concentration and solvent-extraction strategy as factors. For DL, a significant effect of PLGA concentration was observed (*p* < 0.0001), whereas solvent-extraction strategy (*p* = 0.425) and the interaction between the two factors (*p* = 0.276) were not significant. For EE, PLGA concentration also had a significant effect (*p* < 0.0001), whereas solvent-extraction strategy (*p* = 0.383) and the interaction (*p* = 0.364) were not significant.

Having established the effects of solvent-extraction strategy on particle-size uniformity, we next examined whether DEX incorporation was affected by solvent-extraction strategy or PLGA concentration. Drug loading (DL) and encapsulation efficiency (EE) are summarized in Table 2 and Figure 5. DL increased with increasing PLGA concentration for both DSE and SSE (Figure 5a). Two-way ANOVA showed a significant effect of PLGA concentration on DL (*p* < 0.0001), whereas neither solvent-extraction strategy (*p* = 0.425) nor the interaction between both factors (*p* = 0.276) was significant. A similar concentration-dependent pattern was observed for EE (Figure 5b), with a significant effect of PLGA concentration (*p* < 0.0001), but no significant effect of solvent-extraction strategy (*p* = 0.383) or interaction between both factors (*p* = 0.364). Thus, mean DEX incorporation was predominantly governed by PLGA concentration rather than solvent-extraction strategy. Because the DEX-to-PLGA ratio was maintained constant across formulations, these concentration-dependent differences cannot be attributed to differences in the relative initial DEX content with respect to PLGA.

This concentration-dependent behavior is consistent with previous reports suggesting that increasing PLGA concentration enhances drug retention during solvent extraction, presumably by increasing dispersed-phase viscosity and reducing drug diffusion into the external aqueous phase [38,39]. Moreover, polymer concentration can influence solvent exchange and PLGA precipitation kinetics, thereby affecting the time available for drug redistribution before entrapment within the precipitating polymer matrix [37].

In conventional emulsion-based methods, increasing PLGA concentration can also increase particle diameter, which may further reduce drug partitioning into the external aqueous phase by decreasing the surface-area-to-volume ratio of the dispersed phase [33,36]. In the present study, however, processing conditions were adjusted to obtain comparable final particle diameters across PLGA concentrations (Figure 2). Consequently, the observed concentration-dependent increase in DL and EE cannot readily be attributed to differences in final particle size, supporting a more direct contribution of formulation composition and the associated solvent extraction and polymer-precipitation processes [38,40]. Thus, while our previous work demonstrated concentration-dependent effects on droplet shrinkage during DSE of blank PLGA formulations [26], the present results show that extending DSE to DEX-loaded microparticles does not adversely affect drug incorporation relative to SSE.

Although the solvent-extraction strategy had no significant effect on mean DL or EE, the batch-level data revealed differences in variability between processing conditions (Figure 5 and Table 2). At 10% (*w*/*v*) PLGA, the SSE formulation showed greater dispersion than DSE for both DL and EE (SD: 1.45 vs. 0.76 percentage points for DL and 8.67 vs. 4.55 percentage points for EE, respectively). The greatest absolute batch-to-batch variability was observed for the 5% (*w*/*v*) SSE formulation, with SDs of 3.05 and 16.08 percentage points for DL and EE, respectively. A mechanistic interpretation of these differences requires consideration of the coupled solvent extraction, polymer-precipitation, and drug-redistribution processes occurring during microparticle formation. Polymer concentration has previously been shown to alter solvent exchange and precipitation behavior during PLGA microparticle formation, resulting in differences in the developing polymer structure and drug distribution [41]. During DSE, solvent extraction and particle shrinkage proceed while individual droplets are continuously transported through a defined microfluidic pathway, providing a more controlled mass-transfer environment, as demonstrated previously for this platform [26]. In SSE, freshly generated droplets are instead transferred directly into the external collection medium, where most of the subsequent solvent extraction and droplet-to-particle transformation occurs. Such differences could contribute to the greater DL and EE dispersion observed for the 10% and 5% (*w*/*v*) SSE formulations. However, because this difference was small at 2.5% (*w*/*v*) PLGA, the effect cannot be attributed to the solvent-extraction strategy alone and appears to depend on its interaction with formulation composition. That the 5% (*w*/*v*) formulations showed particularly high variability is noteworthy in this context. Both DSE and SSE showed appreciable batch-to-batch variation at this concentration, but the highest level (in an absolute sense) was in SSE. It is therefore likely that 5% (*w*/*v*) PLGA was a process-sensitive formulation under the specific conditions studied rather than a critical polymer concentration in a generic sense. The sensitivity of this formulation may arise from the relative balance of solvent extraction and development of the polymer-rich matrix such that relatively small differences in solvent extraction and polymer-precipitation conditions can translate into differences in drug retention. The present experiments did not directly measure intraparticle DEX redistribution or local solvent concentrations during microparticle formation; therefore, this mechanism should be regarded as an interpretation of the observed batch-level behavior rather than direct evidence of its origin.

### 3.4. Solvent-Extraction Strategy and PLGA Concentration Modulate Microparticle Morphology

**Figure 6:**
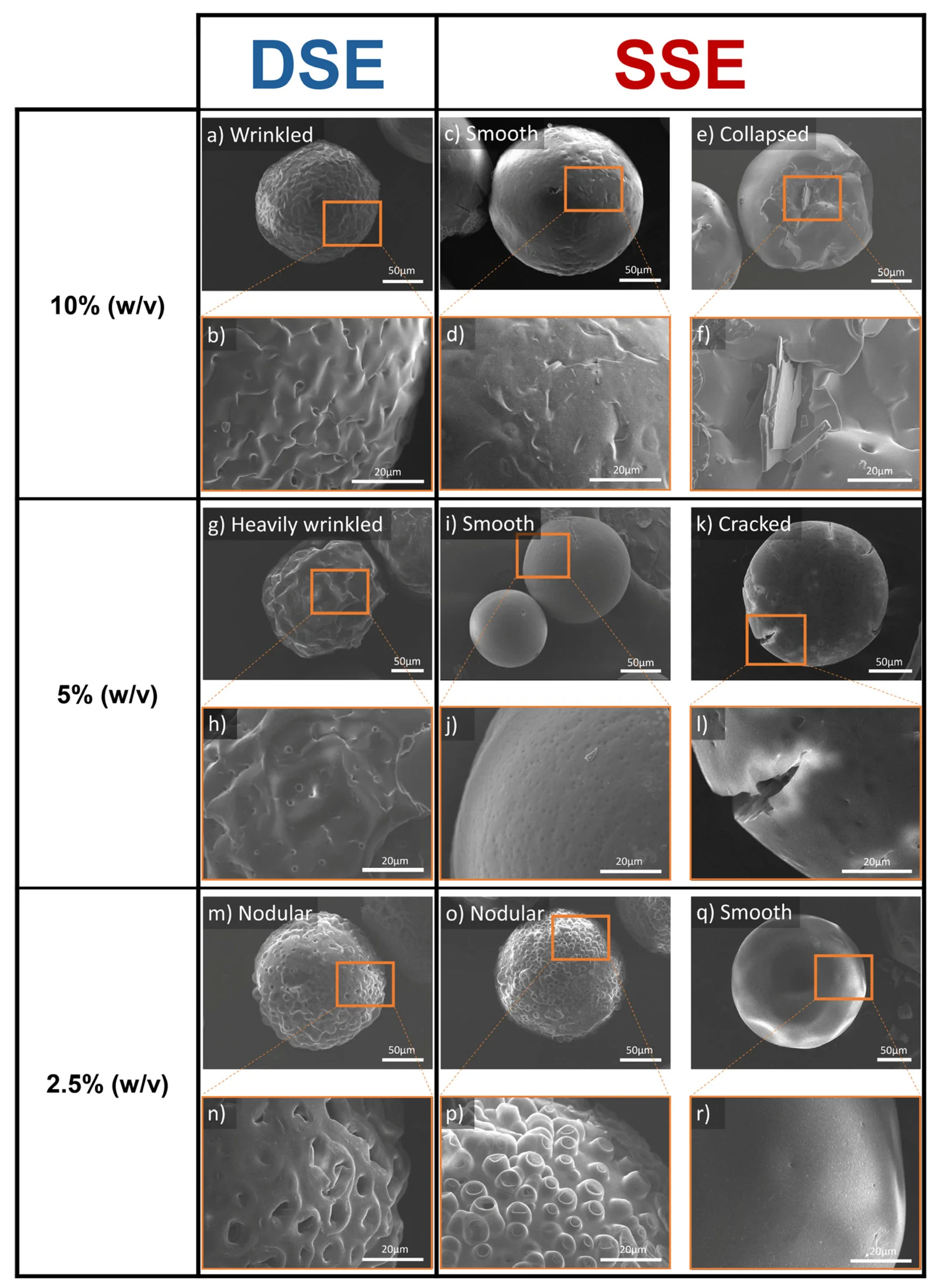
Surface morphology of DEX-loaded PLGA microparticles produced by dynamic solvent extraction (DSE) and static solvent extraction (SSE). Scanning electron microscopy (SEM) images show microparticles prepared at 10% (*w*/*v*) PLGA (**a**–**f**), 5% (*w*/*v*) PLGA (**g**–**l**), and 2.5% (*w*/*v*) PLGA (**m**–**r**). For each formulation, representative particle images are shown together with corresponding higher-magnification surface details. Orange boxes indicate the regions shown in the corresponding higher-magnification images directly below. DSE particles are shown in (**a**,**b**), (**g**,**h**), and (**m**,**n**), whereas SSE particles are shown in (**c**–**f**), (**i**–**l**), and (**o**–**r**) for 10, 5, and 2.5% (*w*/*v*) PLGA, respectively. The additional SSE image pairs illustrate formulation-dependent morphological heterogeneity observed within the corresponding conditions. Representative particle images were acquired at 450–600× magnification and surface-detail images at 1700–2000× magnification. Scale bars are indicated in each image.

Having established that drug incorporation was primarily governed by PLGA concentration rather than solvent-extraction strategy, we next examined whether the solvent-extraction strategy altered microparticle morphology and structural integrity. The morphology of DEX-loaded PLGA microparticles produced using dynamic solvent extraction (DSE) and static solvent extraction (SSE) is shown in Figure 6. Microparticles were successfully obtained at all PLGA concentrations, but their surface morphology differed according to both PLGA concentration and solvent-extraction strategy.

At 10% (*w*/*v*) PLGA, DSE particles exhibited a relatively uniform but wrinkled surface with fine corrugations spread over the surface of the particles (Figure 6a,b). In contrast, SSE particles appeared predominantly smooth and dense overall (Figure 6c,d), even though occasional collapsed surfaces (Figure 6e,f) were noted.

**Figure A1:**
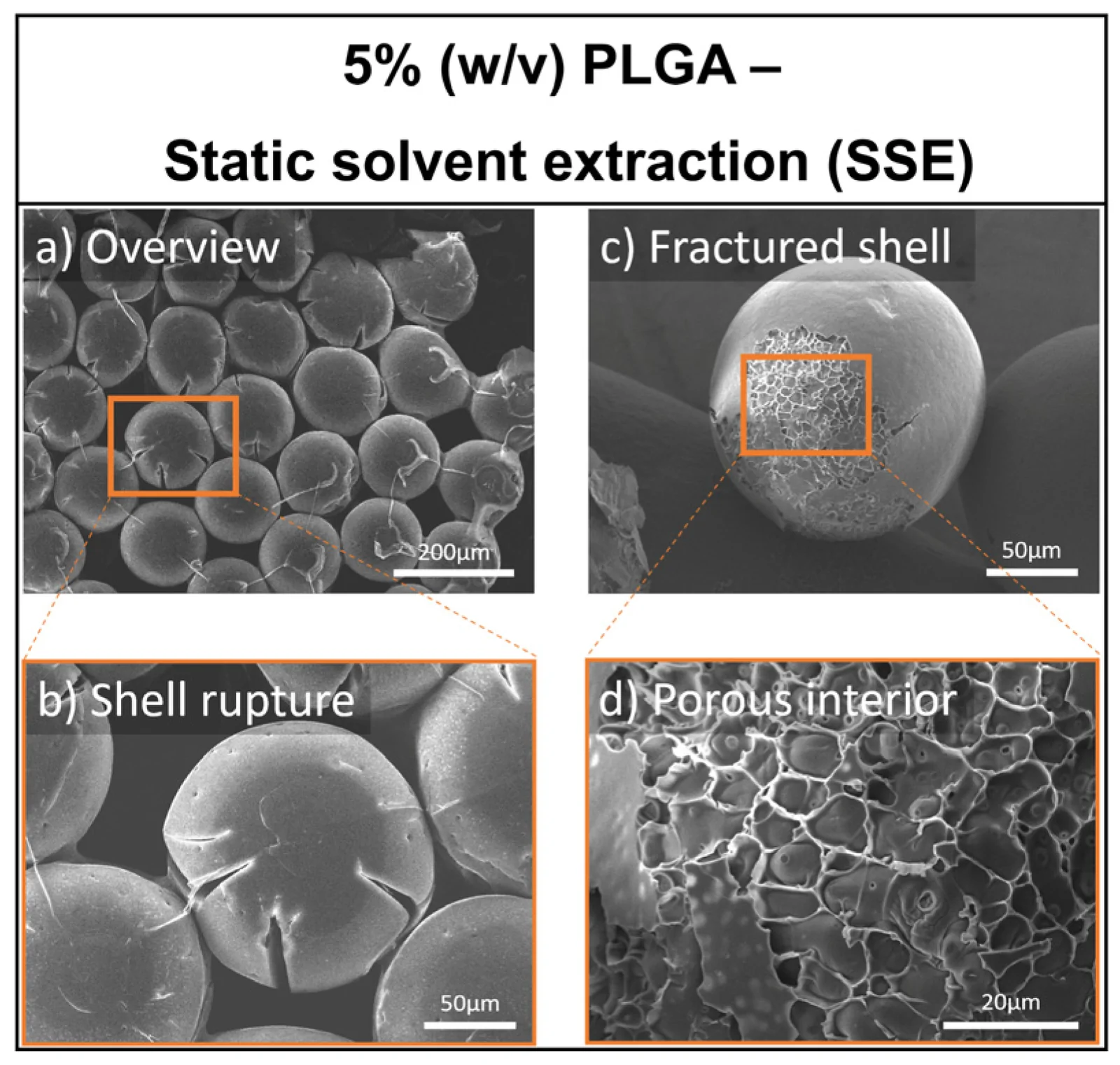
Scanning electron microscopy (SEM) images of DEX-loaded PLGA microparticles prepared at 5% (*w*/*v*) PLGA using static solvent extraction (SSE), illustrating examples of the structural defects observed for this formulation. (**a**) Overview image showing multiple particles with visible surface defects. (**b**) Representative particle exhibiting localized shell rupture. (**c**) Representative fractured particle showing pronounced shell disruption and exposure of the internal structure. (**d**) Higher-magnification image of the fractured region indicated in (**c**), revealing a porous internal structure beneath the particle shell. Orange boxes indicate regions selected for higher magnification imaging. Scale bars are indicated in each image.

At 5% (*w*/*v*) PLGA, the differences became more pronounced. DSE particles exhibited extensive surface wrinkling (Figure 6g,h), whereas SSE particles were generally smooth but frequently displayed localized surface cracks (Figure 6i–l). In some particles, these defects extended into the particle interior, exposing a sponge-like internal structure (Figure A1a–d).

At 2.5% (*w*/*v*) PLGA, DSE microparticles displayed a nodular surface with fine protrusions (Figure 6m,n), whereas SSE microparticles exhibited greater morphological heterogeneity, including nodular (Figure 6o,p) and smoother, more compact (Figure 6q,r) surface morphologies.

The comparatively consistent morphology observed among DSE particles is consistent with the more defined solvent-extraction conditions provided by the microfluidic device. During DSE, droplets are transported through the same defined microfluidic pathway, providing controlled residence time and mass-transfer conditions during solvent extraction and droplet-to-particle transformation [26]. These conditions may reduce droplet-to-droplet variation in solvent extraction and shrinkage compared with SSE, where freshly generated droplets are transferred to the external aqueous phase and undergo most of their subsequent transformation in bulk [26]. Controlled solvent extraction during DSE may also influence the development of a polymer-rich surface layer, as previously described during phase inversion and solvent-extraction-driven formation of PLGA microparticles [23,24,42]. Continued solvent loss and matrix contraction could subsequently generate compressive stresses that are accommodated through surface wrinkling rather than shell rupture [4]. This interpretation is consistent with our previous report, in which DSE likewise produced more uniform, wrinkled PLGA microparticles [26].

The consequences of the different solvent-extraction environments were most apparent under SSE, where the prolonged droplet-to-particle transformation in the bulk collection medium was associated with formulation-dependent morphological responses. The 10% (*w*/*v*) SSE formulation predominantly retained a smooth and continuous surface despite particle contraction, whereas the 2.5% (*w*/*v*) SSE formulation exhibited heterogeneous surface morphology but did not show the frequent cracking observed at 5% (*w*/*v*) PLGA. The 5% (*w*/*v*) SSE formulation therefore appears to represent a particularly process-sensitive condition. Such formulation dependence is consistent with previous observations that polymer concentration can alter solvent exchange and precipitation behavior during microparticle formation and consequently influence the particle structure developing during solvent extraction and polymer precipitation [24,41,43]. One possibility is that the development of the outer polymer-rich layer and continued solvent removal from the particle interior occurred at inadequately matched rates, resulting in non-uniform accommodation of the consequent volume contraction. Continued contraction would then impose stress on the developing surface, which would be relieved in some particles through cracking/fracture instead of through continuous deformation. This interpretation is consistent with the presence of co-occurring smooth particles, cracked surfaces, and fractured shells within the 5% (*w*/*v*) SSE formulation. However, this proposed mechanism remains interpretative because the kinetics of polymer precipitation and the mechanical properties of the developing particle structure were not directly measured.

Overall, these findings suggest that the solvent-extraction strategy can change surface morphology and structural integrity, and that the extent and nature of this change depend on PLGA concentration. The greatest loss of structural integrity was observed for the 5% (*w*/*v*) PLGA SSE formulation among the conditions examined.

### 3.5. Burst Release Trends Differ with Solvent-Extraction Strategy, While Long-Term Release Broadly Converges

**Figure 7:**
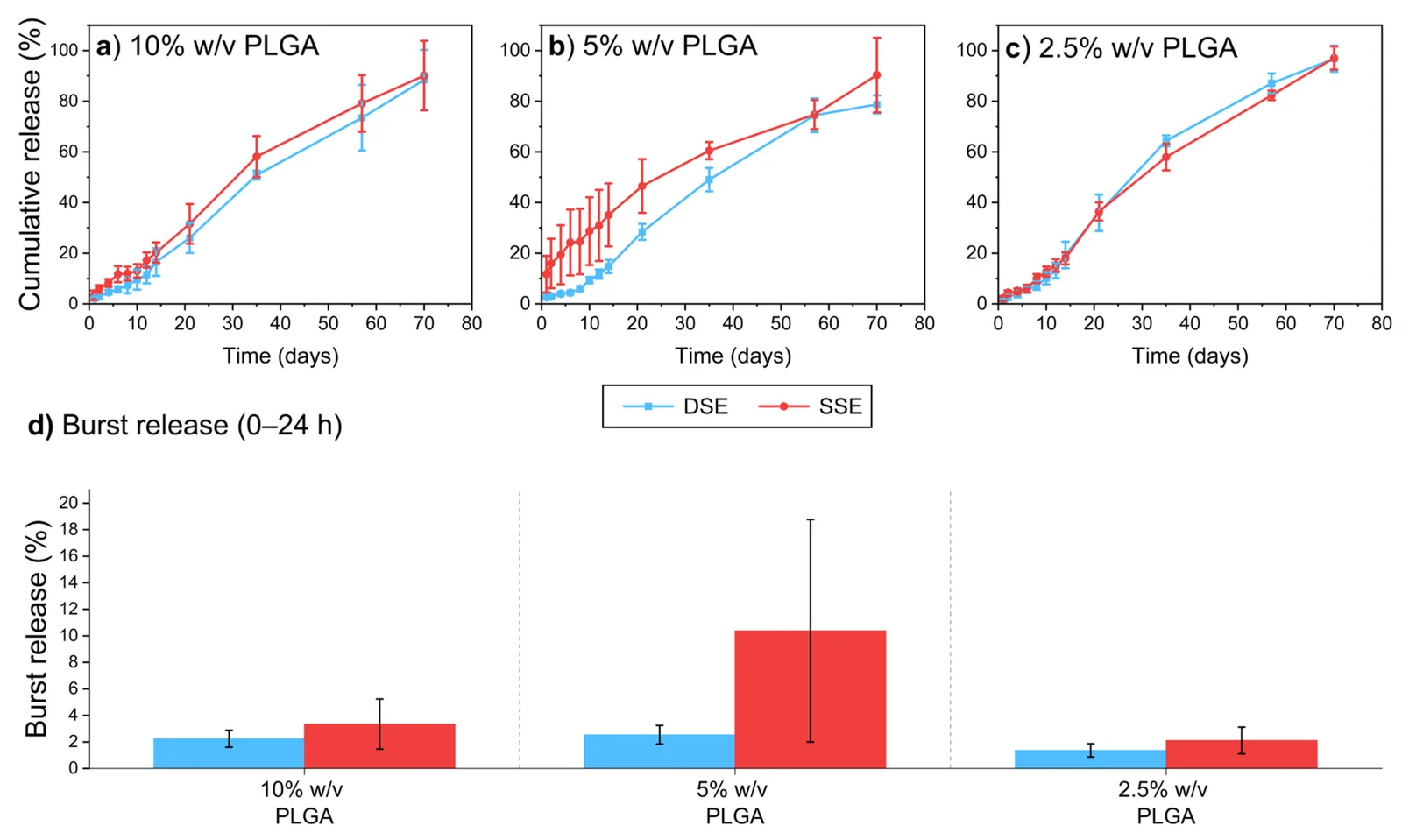
Influence of solvent-extraction strategy on dexamethasone release from PLGA microparticles prepared at (**a**) 10% (*w*/*v*), (**b**) 5% (*w*/*v*), and (**c**) 2.5% (*w*/*v*) PLGA. Cumulative in vitro release profiles were monitored over 70 days for microparticles prepared by dynamic solvent extraction (DSE) and static solvent extraction (SSE). (**d**) Initial burst release during the first 24 h. Data are presented as mean ± standard deviation (SD) from independent production batches (n = 3 for all formulations, except 10% (*w*/*v*) SSE, for which n = 4). The effects of PLGA concentration and solvent-extraction strategy on 24 h burst release were evaluated by two-way ANOVA; neither PLGA concentration (*p* = 0.146) nor solvent-extraction strategy (*p* = 0.106) showed a statistically significant overall effect.

Because particle morphology and shell integrity are relevant to water penetration and drug diffusion, we next evaluated whether the observed morphological differences were associated with differences in DEX release behavior. Monitoring of in vitro DEX release over 70 days showed sustained release from all formulations, characterized by a modest initial burst followed by prolonged release over the remainder of the study period (Figure 7a–c). Despite differences in solvent-extraction strategy and PLGA concentration, the long-term release profiles showed broadly comparable sustained-release behavior.

The most apparent differences between DSE and SSE occurred during the initial 24 h release period (Figure 7d). DSE formulations showed low mean burst release across all PLGA concentrations (1.4–2.7%), whereas the corresponding SSE formulations consistently showed numerically higher values (2.1–11.7%). However, two-way ANOVA revealed no statistically significant overall effect of either PLGA concentration (*p* = 0.146) or solvent-extraction strategy (*p* = 0.106) on 24 h burst release. The numerical difference between DSE and SSE was most pronounced at 5% (*w*/*v*) PLGA, where mean burst release was approximately fourfold higher for SSE than for DSE. The 5% (*w*/*v*) SSE formulation also showed the greatest numerical batch-to-batch variability in burst release (Table 3).

Notably, the largest numerical difference in burst release corresponded with the greatest morphological differences between particles resulting from the two solvent-extraction strategies. Scanning electron microscopy showed frequent surface cracking and shell disruption for 5% (*w*/*v*) SSE particles, whereas the corresponding DSE formulation featured more consistent particles without similarly evident structural defects. Such structural heterogeneity may facilitate water penetration and drug diffusion, consistent with previous reports linking microstructural heterogeneity and surface-associated porosity to burst release from PLGA systems [39,40]. In contrast, the 10% and 2.5% (*w*/*v*) PLGA formulations had a much smaller difference in burst release between DSE and SSE, despite morphological heterogeneity being evident in the corresponding SSE formulations.

Apart from morphology, particle size and particle-size distribution may influence release behavior through their effects on surface-area-to-volume ratio and diffusion distance. However, particle-size differences are unlikely to provide an explanation for the formulation-specific release trends observed here, as DSE and SSE formulations were intentionally produced within a comparable final particle-size range. In particular, the 2.5% (*w*/*v*) SSE formulation showed an appreciably broader particle-size distribution than its DSE counterpart, but only a relatively small difference in 24 h burst release. Conversely, the largest numerical difference in burst release occurred at 5% (*w*/*v*) PLGA, where pronounced cracks and shell disruption were observed in the SSE particles. Thus, although a contribution of particle-size variability cannot be excluded, the combined observations suggest that the 5% (*w*/*v*) formulation was particularly sensitive to the solvent-extraction environment under the conditions investigated. Importantly, because the overall effect of solvent-extraction strategy on burst release did not reach statistical significance, these observations should be interpreted as formulation-specific trends rather than evidence of a generalized effect of DSE on burst release.

Release profiles were further fitted to zero-order and Korsmeyer–Peppas models to summarize their kinetic behavior rather than to assign a single release mechanism (Table 4). Most formulations were reasonably described by both models. The 5% (*w*/*v*) SSE formulation was a notable exception, showing poor and highly variable zero-order fitting while remaining well described by the Korsmeyer–Peppas model. This deviation coincided with the formulation showing the greatest morphological heterogeneity and the largest variability in initial burst release. Given the empirical nature of these models and the limited number of independent batches, however, the fitted parameters were considered descriptive of release behavior and were not used to assign a specific release mechanism.

Overall, DSE formulations consistently showed numerically lower 24 h burst release than their corresponding SSE formulations, although the overall effect of solvent-extraction strategy was not statistically significant. The magnitude and variability of this difference were formulation-dependent and were most pronounced at 5% (*w*/*v*) PLGA, coinciding with the greatest morphological heterogeneity observed under SSE. At longer times, all formulations exhibited broadly comparable sustained-release behavior. Together, these findings indicate that differences associated with the solvent-extraction environment were most apparent during the early phase of DEX release, while DSE remained compatible with sustained DEX release over the 70-day study period.

## 4. Conclusions

This work identifies the solvent-extraction environment as an important process parameter in the microfluidic production of dexamethasone-loaded PLGA microparticles. By comparing dynamic solvent extraction (DSE) with static solvent extraction (SSE) under matched droplet-generation conditions and with comparable final particle sizes, the contribution of the subsequent solvent-extraction-driven droplet-to-particle transition to microparticle quality could be evaluated independently of major differences in final particle size. DSE better preserved particle-size uniformity across the investigated PLGA concentrations, as reflected by the significantly lower particle-size CV compared with SSE. DSE particles also exhibited comparatively consistent morphologies, whereas SSE resulted in more formulation-dependent morphological heterogeneity.

In contrast, mean DEX incorporation was governed predominantly by PLGA concentration rather than solvent-extraction strategy, with both DL and EE increasing with increasing PLGA concentration and no significant overall effect of DSE versus SSE. Differences in batch-to-batch variability were more formulation dependent: greater dispersion under SSE was particularly apparent at 10% and 5% (*w*/*v*) PLGA, whereas differences between the two extraction strategies were comparatively modest at 2.5% (*w*/*v*) PLGA. The 5% (*w*/*v*) formulation emerged as the most process-sensitive condition, with SSE producing frequent surface cracks and structural defects together with the greatest variability in DL, EE, and initial burst release.

All formulations maintained sustained DEX release over the 70-day study period, and their long-term release profiles broadly converged. DSE nevertheless produced consistently lower numerical 24 h burst release than SSE across the investigated PLGA concentrations, with the largest difference observed at 5% (*w*/*v*) PLGA. As the overall effect of the solvent-extraction strategy on burst release did not reach statistical significance, this finding should be interpreted as a formulation-dependent trend rather than a generalized effect of DSE.

Taken together, these findings demonstrate that the dynamic solvent-extraction strategy can improve the preservation of particle-size uniformity without adversely affecting drug incorporation or sustained-release performance. The pronounced sensitivity of the 5% (*w*/*v*) formulation further highlights that the effect of the solvent-extraction strategy depends on formulation composition. Overall, these findings provide a process-level foundation for extending control beyond microfluidic droplet generation to the subsequent droplet-to-particle transition in scalable manufacturing strategies for sustained-release PLGA microparticles.

## Figures and Tables

**Table 1:** Summary statistics describing particle size reproducibility of DEX-loaded PLGA microparticles produced by dynamic solvent extraction (DSE) and static solvent extraction (SSE) at different polymer concentrations. The coefficient of variation (CV) of particle diameter is reported as mean ± standard deviation (SD) and median with interquartile range (IQR) to characterize both average variability and batch-to-batch consistency. Each value represents an independent production batch (n = 5 per formulation).

Group	Mean CV ± SD	Median CV (Q1–Q3)
10% DSE	5.17 ± 1.25	5.49 (5.08–5.92)
10% SSE	9.07 ± 5.24	6.38 (6.15–11.22)
5% DSE	6.09 ± 2.16	7.29 (4.62–7.32)
5% SSE	8.98 ± 5.26	7.38 (6.35–10.82)
2.5% DSE	5.79 ± 1.90	5.68 (5.00–5.88)
2.5% SSE	10.07 ± 2.53	10.45 (9.29–10.56)

**Table 2:** Summary statistics for DEX incorporation in PLGA microparticles produced by dynamic solvent extraction (DSE) and static solvent extraction (SSE) at different polymer concentrations. Drug loading (DL) and encapsulation efficiency (EE) are reported as mean ± standard deviation (SD) and as median with interquartile range (IQR) to describe both central tendency and batch-to-batch variability. Each value represents an independent production batch (n = 5 per formulation).

Group	Mean DL ± SD	Median DL (Q1–Q3)	Mean EE ± SD	Median EE (Q1–Q3)
10% DSE	12.91 ± 0.76	12.40 (12.32–13.57)	77.54 ± 4.55	74.38 (73.98–81.61)
10% SSE	11.28 ± 1.45	11.35 (11.08–11.97)	67.99 ± 8.67	68.66 (67.02–72.54)
5% DSE	9.23 ± 2.47	9.94 (9.29–10.55)	55.41 ± 15.36	60.16 (55.73–63.29)
5% SSE	7.97 ± 3.05	9.06 (5.06–9.56)	48.08 ± 16.08	54.10 (30.95–59.14)
2.5% DSE	6.29 ± 1.62	6.92 (4.64–7.48)	38.62 ± 9.76	42.39 (27.36–48.46)
2.5% SSE	7.40 ± 1.81	8.15 (7.33–8.45)	43.68 ± 10.84	48.40 (43.13–48.89)

**Table 3:** Summary statistics for the initial burst release (0–24 h) of DEX-loaded PLGA microparticles produced by dynamic solvent extraction (DSE) and static solvent extraction (SSE). Data are presented as mean ± SD and median (Q1–Q3) to capture both average performance and inter-batch reproducibility.

Group	Mean Burst Release ± SD	Median Burst Release (Q1–Q3)
10% DSE	2.24 ± 0.64	2.21 (1.85–2.63)
10% SSE	3.35 ± 1.89	3.55 (3.13–4.87)
5% DSE	2.65 ± 0.30	2.07 (2.04–2.80)
5% SSE	11.72 ± 7.23	6.87 (4.60–14.41)
2.5% DSE	1.37 ± 0.50	1.12 (1.02–1.59)
2.5% SSE	2.11 ± 1.01	2.04 (1.48–2.71)

**Table 4:** Release kinetic modelling parameters for DEX-loaded PLGA microparticles prepared by dynamic solvent extraction (DSE) and static solvent extraction (SSE). Experimental release profiles were fitted to zero-order and Korsmeyer–Peppas (K–P) models. Values represent the mean ± SD of individual batch fits. Adjusted coefficients of determination (R^2^adj) are shown as measures of goodness-of-fit, while the Korsmeyer–Peppas release exponent (n) is reported as a descriptive parameter of release behavior. Day 70 release (%) represents the cumulative DEX released at the end of the 70-day study.

Group	Zero-Order R^2^adj	K-P R^2^adj	K-P n	Day 70 Release (%)
10% DSE	0.971 ± 0.009	0.968 ± 0.012	0.946 ± 0.105	88.4 ± 11.9
10% SSE	0.977 ± 0.011	0.956 ± 0.039	0.808 ± 0.089	90.1 ± 13.7
5% DSE	0.963 ± 0.018	0.938 ± 0.013	0.919 ± 0.072	92.5 ± 1.2
5% SSE	−0.272 ± 1.698	0.971 ± 0.008	0.588 ± 0.250	89.2 ± 14.3
2.5% DSE	0.966 ± 0.011	0.942 ± 0.013	1.076 ± 0.090	96.8 ± 5.2
2.5% SSE	0.986 ± 0.003	0.980 ± 0.008	0.959 ± 0.110	97.1 ± 4.6

## Data Availability

The original contributions presented in this study are included in the article. Further inquiries can be directed to the corresponding authors.

## Abbreviations

The following abbreviations are used in this manuscript:

ANOVA	Analysis of variance
COC	Cyclic olefin copolymer
CQA	Critical quality attribute
CV	Coefficient of variation
DEX	Dexamethasone
DL	Drug loading
DMSO	Dimethyl sulfoxide
DSE	Dynamic solvent extraction
EE	Encapsulation efficiency
EMA	European Medicines Agency
FDA	U.S. Food and Drug Administration
HC	Hixson–Crowell
ID	Inner diameter
IDL	Initial drug loading
K–P	Korsmeyer–Peppas
OD	Outer diameter
PLGA	Poly(lactic-co-glycolic acid)
PBS	Phosphate-buffered saline
PSD	Particle size distribution
PVA	Poly(vinyl alcohol)
Q_c_	Continuous-phase flow rate
Q_d_	Dispersed-phase flow rate
R^2^adj	Adjusted coefficient of determination
SEM	Scanning electron microscopy
SD	Standard deviation
SSE	Static solvent extraction
UV–Vis	Ultraviolet–visible
*w*/*v*	Weight per volume

## Appendix A

**Table A1:** Comparison of kinetic models used to describe DEX release from PLGA microparticles. Values are mean ± SD of individual batch fits.

Group	Zero-Order R^2^adj	First-Order R^2^adj	Higuchi R^2^adj	Hixson–Crowell R^2^adj	K-P R^2^adj	K-P n	Weibull R^2^adj	Weibull β	Day 70 Release (%)
10% DSE	0.97 ± 0.01	0.91 ± 0.07	0.78 ± 0.07	0.94 ± 0.04	0.968 ± 0.012	0.946 ± 0.105	0.94 ± 0.05	1.22 ± 0.18	88.4 ± 11.9
10% SSE	0.97 ± 0.01	0.95 ± 0.03	0.84 ± 0.02	0.94 ± 0.05	0.956 ± 0.039	0.808 ± 0.089	0.98 ± 0.01	1.04 ± 0.02	90.1 ± 13.7
5% DSE	0.96 ± 0.02	0.91 ± 0.04	0.80 ± 0.03	0.96 ± 0.01	0.938 ± 0.013	0.919 ± 0.072	0.95 ± 0.01	1.22 ± 0.07	92.5 ± 1.2
5% SSE	−0.27 ± 1.70	0.43 ± 0.68	0.66 ± 0.32	0.95 ± 0.03	0.971 ± 0.008	0.588 ± 0.250	0.30 ± 0.92	0.74 ± 0.30	89.2 ± 14.3
2.5% DSE	0.97 ± 0.01	0.86 ± 0.10	0.78 ± 0.04	0.94 ± 0.02	0.942 ± 0.013	1.076 ± 0.090	0.97 ± 0.01	1.27 ± 0.04	96.8 ± 5.2
2.5% SSE	0.99 ± 0.01	0.87 ± 0.05	0.81 ± 0.02	0.96 ± 0.01	0.980 ± 0.008	0.959 ± 0.110	0.96 ± 0.01	1.43 ± 0.18	97.1 ± 4.6
